# Profiling of Humoral Immune Response in Typhoid Patients against Differentially Extracted Whole Cell Bacterial Protein Derived from *S. typhi* and *S.* spp

**DOI:** 10.1155/2023/4125588

**Published:** 2023-03-31

**Authors:** Khairunnisa Abdul Lateef Khan, Zainoodin Sheik Abdul Kader

**Affiliations:** Advanced Medical & Dental Institute, Universiti Sains Malaysia, Pulau Pinang 13300, Malaysia

## Abstract

Typhoid fever is a multiorgan infectious disease caused by *Salmonella typhi*. It is transmitted through fecal oral route and can be fatal without proper treatment. Therefore, early diagnosis of typhoid fever is crucial. In the previous study, we have developed TYPHOIDYNE EIA, which showed excellent synergy between the genus conserved and species-specific antigens in the serodiagnosis of typhoid fever. TYPHOIDYNE EIA can effectively detect and differentiate typhoid patients, typhoid vaccinated subjects, healthy subjects, and subjects with other febrile illnesses. Following the successful development of TYPHOIDYNE EIA, in this report, we further characterize the antigenic components of differentially extracted *S. typhi* and *S.* spp recognized by IgM, IgG, and IgA antibody isotypes in typhoid patients and possible typhoid carrier by the western blot (WB) assay. The WB characterization revealed a dynamic pattern of recognition, with significant variations in the number of antigenic bands observed between the differentially extracted arrays of antigens. The reactivity of patient's sera was divided into 3 regions, with region 1 (≥55 kDa) showing the strongest reactivity followed by region 2 (54 kDa–34 kDa) and region 3 (<34 kDa). Overall, the good synergy expressed in these bands suggests the potential role of these proteins in differentiating typhoid patients with possible typhoid carrier. The antigenic bands highlighted in this study are also identified as prospective biomarkers for diagnostic use and vaccine development.

## 1. Introduction

Typhoid fever is an infectious disease that is transmitted through the fecal oral route. It is caused by the bacterial agent, *Salmonella typhi* (*S. typhi*). This typhoid agent can elicit a spectrum of infection outcomes including chronic typhoid, mild infection, relapse after recovery, and passive or acute carrier of typhoid [1]. *Salmonella* carriers are frequently asymptomatic, and they regularly shed the bacteria which cause the spread of the disease [2]. On the other hand, drug resistance against *S. typhi* is increasingly becoming a major cumbersome issue in managing this disease. With the emergence of extensively drug resistance (XDR) strains, there is potential that typhoid fever will become untreatable owing to a lack of antibiotic options [3].

Over the years, numerous studies have been conducted to investigate and discover specific antigenic proteins from various preparations derived from *S. typhi* for the serodiagnosis of typhoid fever. This included characterization of heat shock protein (HSP), capsular-polysaccharide (Vi) antigen, somatic (O) antigen, and flagellar (H) antigen. In this regard, WB analysis of the HSP revealed immunodominant antigenic proteins with molecular weights of 58, 68, and 88 kDa [4]. HSP has been known to play a major role in activating a high degree of humoral immune response in infected individuals [5]. HSP has also been identified as the target of immune response in a wide range of infections [6].

On the other hand, the Vi antigen found in *S. typhi*, *S*. *Paratyphi* C, and *S*. *Dublin* has been shown to aid in escaping innate immune detection and elevate systemic virulence of infection. In 1994, the Vi capsular-polysaccharide vaccine was licensed and has been commercially available throughout the years. However, this vaccine has been shown to elicit paucity of immune response to young children [2, 7]. Therefore, a few other vaccines derived from the Vi antigen which includes Vi-rEPA vaccine and Typbar-TCV have been introduced to elicit a high degree of immune response for better protection from typhoid fever in a variety of age groups, from children to the elderly [8, 9]. However, several limitations such as the short-term effect of these vaccines due to absence of immune memory and the inability of protection against iNTS and other salmonellosis impede the performance of the vaccines [10, 11].

The O and H antigens have been used as a serological diagnostic biomarker in the classical Widal test that was invented in 1896. The Widal test measures the antibody titre against O and H antigens by the agglutination method. Despite the long-term deployment of this method in primary and secondary healthcare facilities in rural settings, the high rate of false positive and false negative cases were observed in endemic areas [12]. Patients that have been previously infected with typhoid or other corelated febrile disease such as coliforms, Shigella, or other salmonellae stimulate production of homogenous antibodies that resulted in a high rate of false positive cases. In addition, vaccinated individuals possessed antigens that stimulated antibody production, resulting in inaccurate diagnosis [13]. These drawbacks in the application of O and H antigens in the Widal test have resulted in a disparate level of its acceptability around the world.

These findings demonstrated a heterogenous antibody response following typhoid infection against different antigens as well as a significant number of drawbacks in the use of single antigen for the diagnosis and prevention of typhoid fever. Therefore, the combination of more than one antigen would be essential to detect the humoral immune response in typhoid patients and contribute to both diagnostic value and vaccine development against typhoid fever. The generation of multiantigens -ased immunoassay may assist in the development of serological assay with improved sensitivity and specificity.

Previously, we have successfully developed TYPHOIDYNE EIA that have successfully detected and differentiated typhoid cases, typhoid vaccinated individuals, typhoid carriers, and healthy individuals. TYPHOIDYNE EIA has effectively highlighted the synergistic effect of the genus conserved and species specific antigens in the serodiagnosis of typhoid fever.

Therefore, in this present study, we aim to further characterize the WCP, CSP, and sdWCP derived from *S. typhi* and *S.* spp using WB analysis and highlight the significance of multiple antigens inclusion in the development of serodiagnosis immunoassay. The WB analysis was carried out to assess the presence of the specific IgM, IgG, and IgA antibody isotypes response in typhoid patients against the differentially extracted antigens. Subsequently, we performed a comparative analysis to evaluate the degree of immune response and differentiate the reactive bands elicited in the WB profiles of typhoid patients compared to the possible carrier from healthy subjects based on TYPHOIDYNE EIA evaluation in our previous report [14].

## 2. Methods

### 2.1. Serum Sample Collection

The overall profile of the humoral immune response against antigens derived from *S. typhi* and *S.* spp were analyzed by WB assay using representative patient's sera from culture and serology positive typhoid cases. Healthy subjects that were categorized as carrier in the previous report were included in this study [14]. To perform the WB assay, sera from typhoid patients and healthy subjects were analyzed as pooled sera, respectively. There were two categories of pooled sera used in WB assay to determine recognizable and representative antigenic bands present in differentially extracted protein of *S. typhi* and *S.* spp and to analyze the diversity in humoral immune response between typhoid patients and the possible typhoid carrier. Pool sera designated as PTS was made up of 4 positive IgM, IgG, and IgA. Pooled sera designated as PHPS was made up from 4 sera taken from possible carrier subjects. The categorized pooled sera are tabulated and summarized in Table 1.

### 2.2. Protein Extraction

The differential protein extraction of WCP, CSP, and sdWCP of *S. typhi* and *S.* spp was performed as described previously [14]. For WCP, each bacterial strain was isolated as a single colony from sheep blood agar, inoculated into 10 ml Luria broth, and cultured overnight at 37°C. The broth was centrifuged (10000 rpm, 4°C, and 10 minutes) after three successive overnight subcultures, and the pellet was resuspended in 1X SPB (pH 6.8). After boiling for 5 minutes, the suspension was centrifuged (10000 rpm, 4°C, and 10 minutes). The suspension was precipitated with ethanol and incubated at −20°C overnight. The suspension was then centrifuged (10000 rpm, 4°C, and 10 minutes). The pellet was dissolved in Tris-PMSF and kept at −20°C until further use. For CSP extraction, the bacterial strain was cultured in the same manner as for WCP extraction. The pellet was resuspended in glycine-HCL (pH 2.2) and incubated for 15 minutes at room temperature before being centrifuged (10000 rpm, 4°C, 10 minutes). The pH of the supernatant was lowered to 7.4 before proceeding with ice cold ethanol precipitation. The sdWCP was extracted using the same method as WCP from the pellet left after the CSP extraction.

### 2.3. Western Blot Analysis

The antigens employed for immunoblotting were WCP, CSP, and sdWCP derived from the two strains. The differentially extracted proteins were separated by SDS-PAGE following the method described by Leammli [15]. After the separation of protein by SDS-PAGE, the gel was incubated in Towbin transfer buffer for 15 minutes. The separated components on the gel were electroblotted to 0.45 µm pore size nitrocellulose(NC) membrane. The Bio-Radsemi-dry transblot apparatus was used to perform the electroblotting. The electroblotting condition was set to 20 V for 30 minutes according to manufacturer's instructions. After the transfer to NC membrane was done, the NC membrane was incubated in Ponceau S staining solution for 15 minutes. The reference lane with the protein marker was then cut off before proceeding with western blot analysis. Next, blocking was done with 5% skimmed milk in 1X TBS for one hour. The NC membrane was then incubated with primary antibody (serum diluted 1 : 100 in 1XTBS) for 2 hours followed by washing three times, for 5 minutes each. The NC membrane was then incubated with the secondary antibody (IgM, IgG, and IgA) for another 2 hours before washing with skimmed milk three times, for 5 minutes each. The membrane strips were than incubated for 10 minutes with AP conjugate for optimum colour development. The reaction was stopped by washing with distilled water for 10 minutes.

## 3. Results

### 3.1. Characterization of O9 (Somatic Antigens) and dH (Flagellar Antigens) against WCP, CSP, and sdWCP Derived from *S. typhi* and *S.* spp

**Figure 1:**
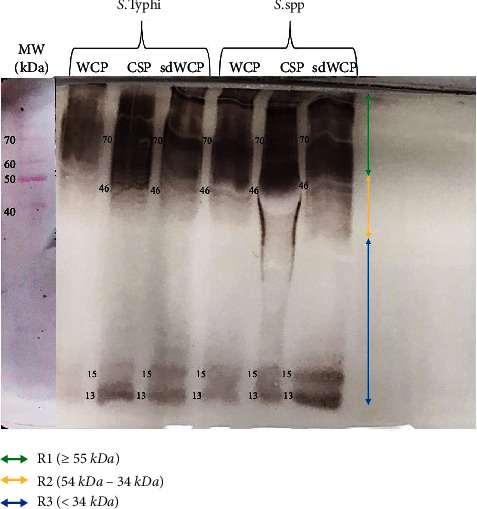
The WB analysis of goat anti-rabbit IgG antibody isotype reactivity against SDS-PAGE-separated antigens from the WCP, CSP, and sdWCP derived from *S. typhi* and *S.* spp. The antigens were probed with *Salmonella* O9 serum. The immunoreactive bands identified in the WB reaction are classified according to the region (top right) and indicated by coloured arrows on the right. The reference lane molecular weight markers stained with Ponceau S are indicated to the most left.

WB assay was performed to identify the location of somatic and flagellar antigens recognized by goat anti-rabbit IgG antibody isotypes in differentially extracted proteins derived from *S. typhi* and *S.* spp. As shown in Figure 1, the reactivity of the WB profile probed with *Salmonella* O9 serum were largely confined in cluster 1 and cluster 2. The zone of smearing bands with a high degree of intensity was observed in cluster 1. The O9 serum also recognized low molecular weight protein bands at the region of 13 kDa and 15 kDa in cluster 3.

**Figure 2:**
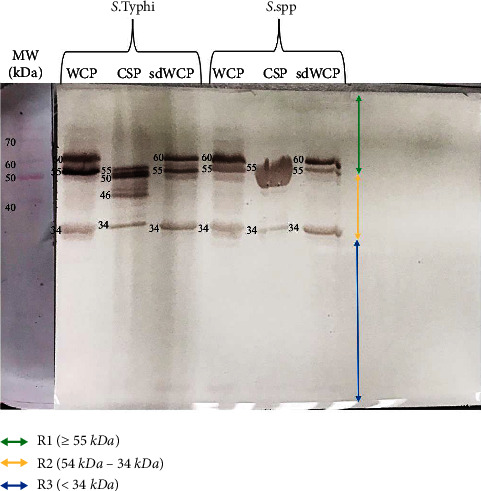
The WB analysis of goat anti-rabbit IgG antibody isotype reactivity against SDS-PAGE-separated antigens from the WCP, CSP, and sdWCP derived from *S. typhi* and *S.* spp. The antigens were probed with *Salmonella* dH serum. The immunoreactive bands identified in the WB reaction are classified according to the region (top right) and indicated by coloured arrows on the right. The reference lane molecular weight markers stained with Ponceau S are indicated to the most left.

On the other hand, well separated and distinct bands were observed in the WB profile in Figure 2. The reactivity against differentially extracted antigens derived from *S. typhi* and *S.* spp probed with dH serum did not show any presence of reactive protein bands in cluster 3. The differentially extracted proteins derived from both *S. typhi* and *S.* spp recognized protein bands at cluster 1 and cluster 2 with two protein bands at the location of 34 kDa and 55 kDa recognized across the six lanes of differentially extracted antigens derived from *S. typhi* and *S.* spp.

### 3.2. Characterization of Antibody Isotypes Response in PTS against WCP, CSP, and sdWCP Derived from *S. typhi* and *S.* spp

**Figure 3:**
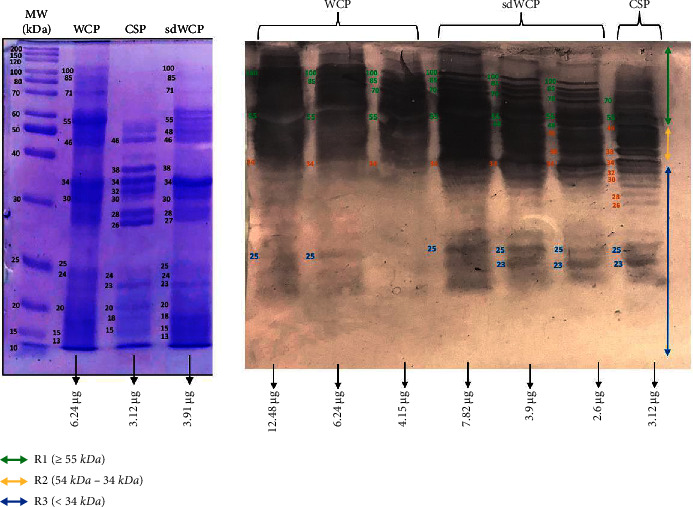
The WB analysis of IgM antibody isotype reactivity against SDS-PAGE-separated antigens derived from WCP, CSP, and sdWCP of *S. typhi* against PTS. The immunoreactive bands identified in the WB reaction are classified according to the region (top right) and indicated by coloured arrows on the right. The reference lane molecular weight markers along with the protein profile stained with Coomassie are indicated to the most left.

**Figure 4:**
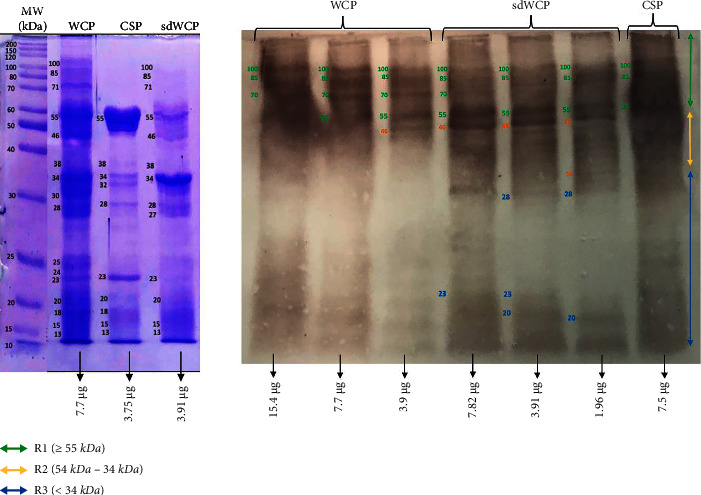
The WB analysis of IgM antibody isotype reactivity against SDS-PAGE-separated antigens derived from WCP, CSP, and sdWCP of *S.* spp against PTS. The immunoreactive bands identified in the WB reaction are classified according to the region (top right) and indicated by coloured arrows on the right. The reference lane molecular weight markers along with the protein profile stained with Coomassie are indicated to the most left.

WB analysis was performed to determine the three main antibody isotypes responses in the panel of pooled sera from typhoid patients designated as PTS against the differentially extracted antigens derived from *S. typhi* and *S.* spp. As shown in Figures 3 and 4, the IgM WB profile of reactive bands recognized by differentially extracted *S. typhi* antigens was closely similar to the WB profile of reactive bands detected by differentially extracted *S.* spp antigens. The IgM WB profiles against *S. typhi* and *S.* spp antigens showed the recognition of reactive bands in cluster 1, cluster 2, and cluster 3 at different degrees of intensity.

**Figure 5:**
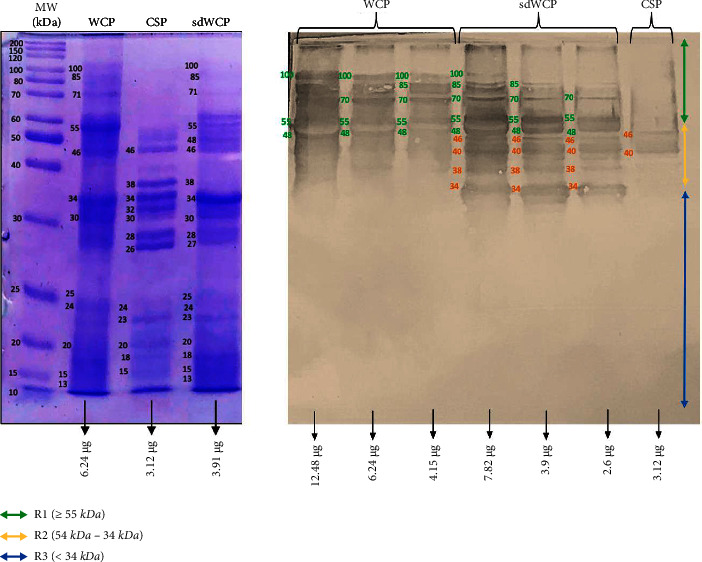
The WB analysis of IgG antibody isotype reactivity against SDS-PAGE-separated antigens from the WCP, CSP, and sdWCP of *S. typhi* against PTS. The immunoreactive bands identified in the WB reaction are classified according to the region (top right) and indicated by coloured arrows on the right. The reference lane molecular weight markers along with the protein profile stained with Coomassie are indicated to the most left.

**Figure 6:**
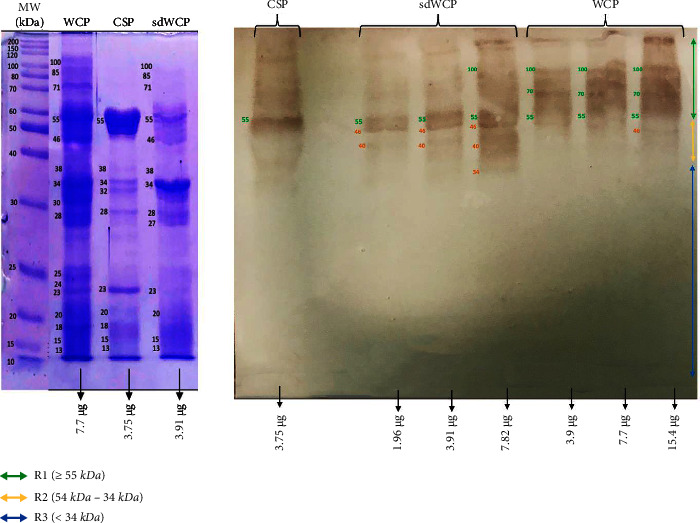
The WB analysis of IgG antibody isotype reactivity against SDS-PAGE-separated antigens from the WCP, CSP, and sdWCP of *S.* spp against PTS. The immunoreactive bands identified in the WB reaction are classified according to the region (top right) and indicated by coloured arrows on the right. The reference lane molecular weight markers along with the protein profile stained with Coomassie are indicated to the most left.

**Figure 7:**
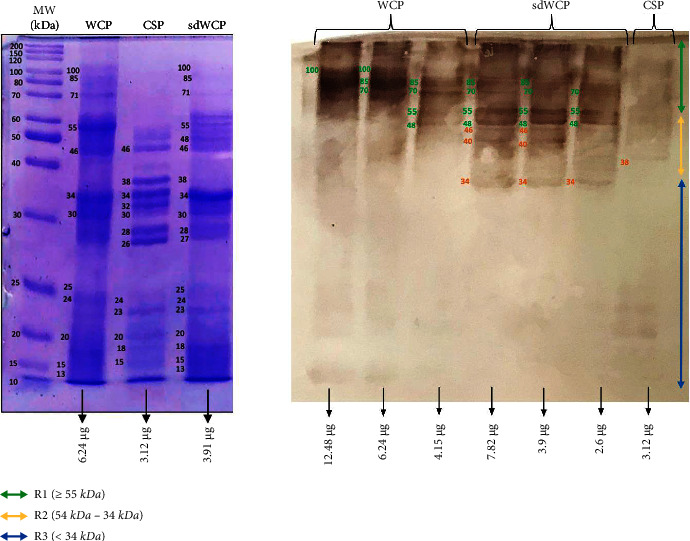
The WB analysis of IgA antibody isotype reactivity against SDS-PAGE-separated antigens from the WCP, CSP, and sdWCP of *S. typhi* against PTS. The immunoreactive bands identified in the WB reaction are classified according to the region (top right) and indicated by coloured arrows on the right. The reference lane molecular weight markers along with the protein profile stained with Coomassie are indicated to the most left.

**Figure 8:**
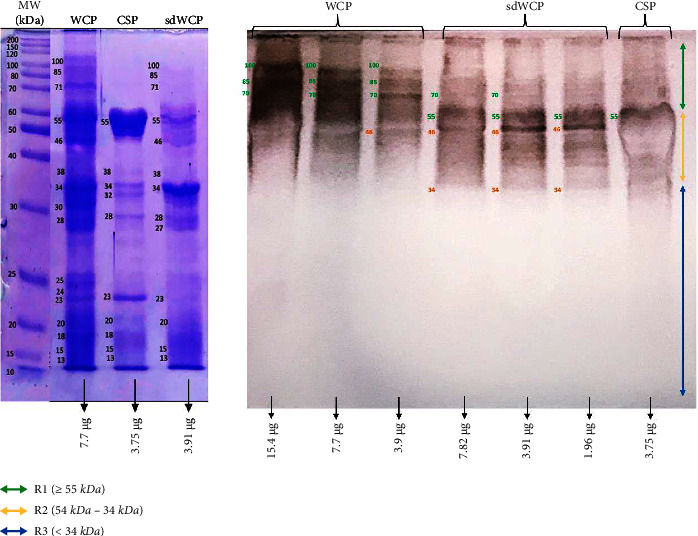
The WB analysis of IgA antibody isotype reactivity against SDS-PAGE-separated antigens from the WCP, CSP, and sdWCP of *S.* spp against PTS. The immunoreactive bands identified in the WB reaction are classified according to the region (top right) and indicated by coloured arrows on the right. The reference lane molecular weight markers along with the protein profile stained with Coomassie are indicated to the most left.

On the other hand, the IgG and IgA WB profiles against differentially extracted *S. typhi* and *S.* spp antigens did not show any presence of protein bands in cluster 3. The WB reactivity studied on the PTS serum pool against IgG and IgA antibody isotypes are shown in Figures 5–8. The WB profile of IgG and IgA antibody isotypes against differentially extracted *S. typhi* antigens was relatively similar to differentially extracted *S.* spp antigens. However, the degree of intensity in the IgG and IgA WB profiles elicited by differentially extracted *S.* spp antigens was lower than differentially extracted *S. typhi* antigens.

### 3.3. Characterization of Antibody Isotypes Responses in PHPS against WCP, CSP, and sdWCP Derived from *S. typhi* and *S.* spp

**Figure 9:**
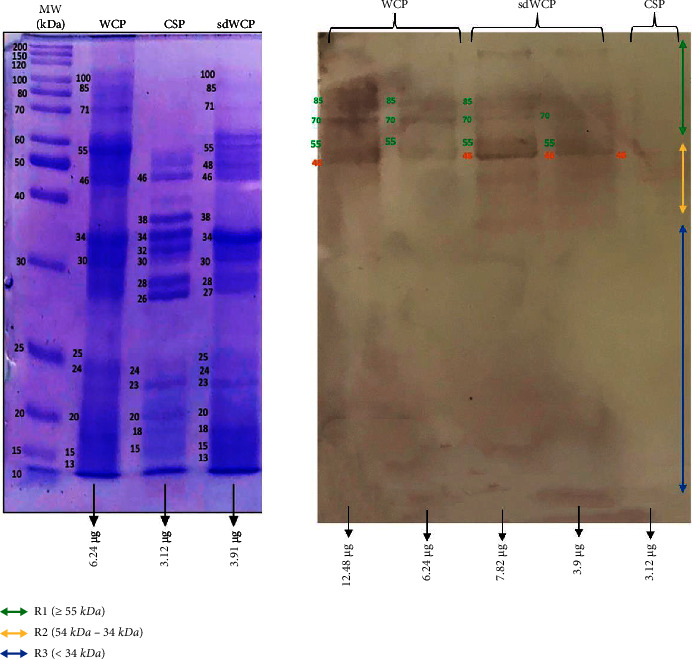
The WB analysis of IgM antibody isotype reactivity against SDS-PAGE-separated antigens from the WCP, CSP, and sdWCP of *S. typhi* against PHPS. The immunoreactive bands identified in the WB reaction are classified according to the region (top right) and indicated by coloured arrows on the right. The reference lane molecular weight markers along with the protein profile stained with coomassie are indicated to the most left.

**Figure 10:**
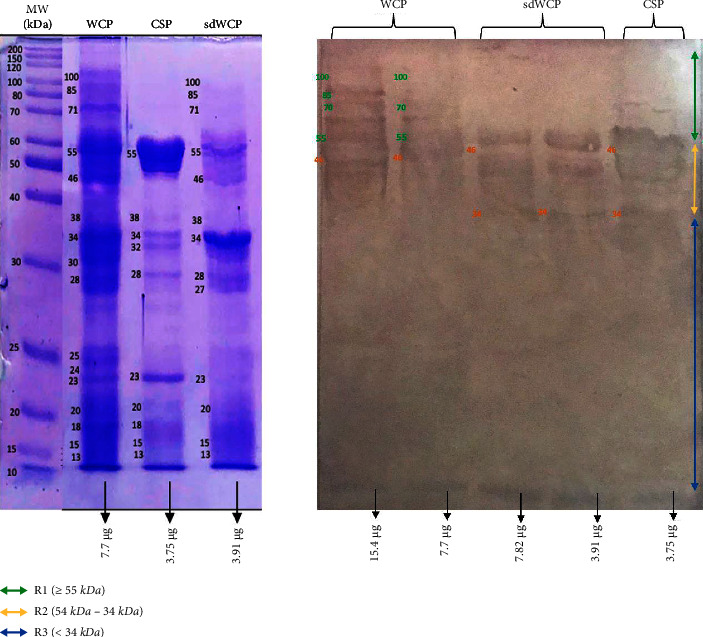
The WB analysis of IgM antibody isotype reactivity against SDS-PAGE separated antigens from the WCP, CSP and sdWCP of *S.* spp against PHPS. The immunoreactive bands identified in the WB reaction are classified according to the region (top right) and indicated by coloured arrows on the right. The reference lane molecular weight markers along with the protein profile stained with Coomassie are indicated to the left most.

**Figure 11:**
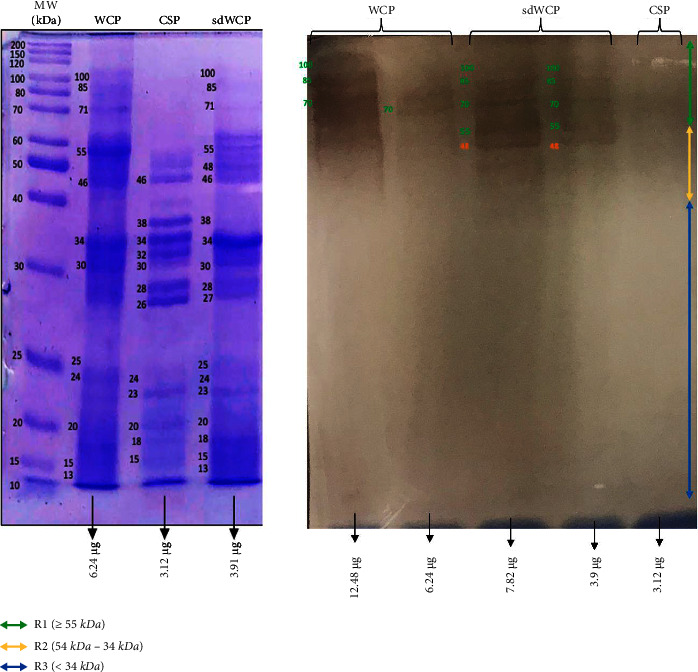
The WB analysis of IgG antibody isotype reactivity against SDS-PAGE-separated antigens from the WCP, CSP, and sdWCP of *S. typhi* against PHPS. The immunoreactive bands identified in the WB reaction are classified according to the region (top right) and indicated by coloured arrows on the right. The reference lane molecular weight markers along with the protein profile stained with Coomassie are indicated to the left most.

**Figure 12:**
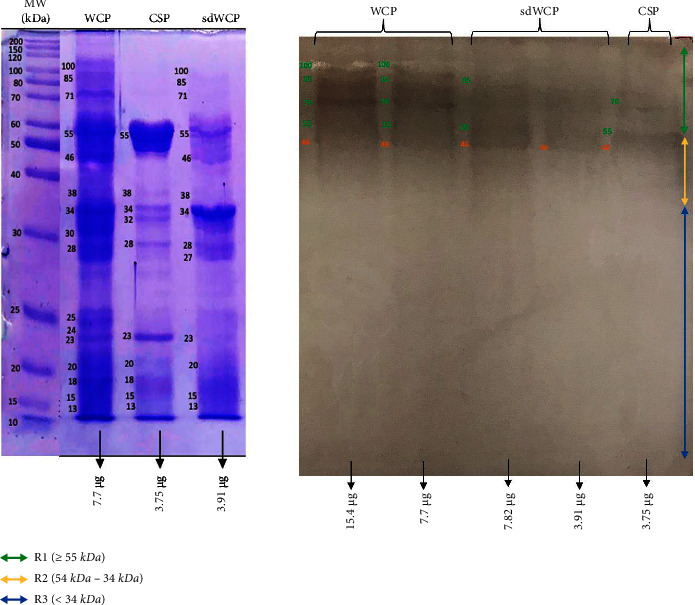
The WB analysis of IgG antibody isotype reactivity against SDS-PAGE-separated antigens from the WCP, CSP, and sdWCP of *S.* spp against PHPS. The immunoreactive bands identified in the WB reaction are classified according to the region (top right) and indicated by coloured arrows on the right. The reference lane molecular weight markers along with the protein profile stained with Coomassie are indicated to the left most.

**Figure 13:**
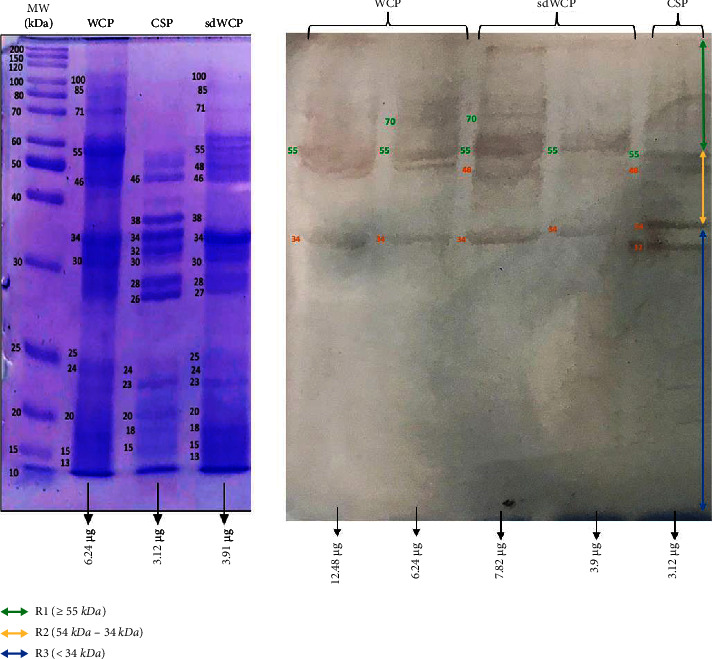
The WB analysis of IgA antibody isotype reactivity against SDS-PAGE-separated antigens from the WCP, CSP, and sdWCP of *S. typhi* against PHPS. The immunoreactive bands identified in the WB reaction are classified according to the region (top right) and indicated by coloured arrows on the right. The reference lane molecular weight markers along with the protein profile stained with Coomassie are indicated to the left most.

**Figure 14:**
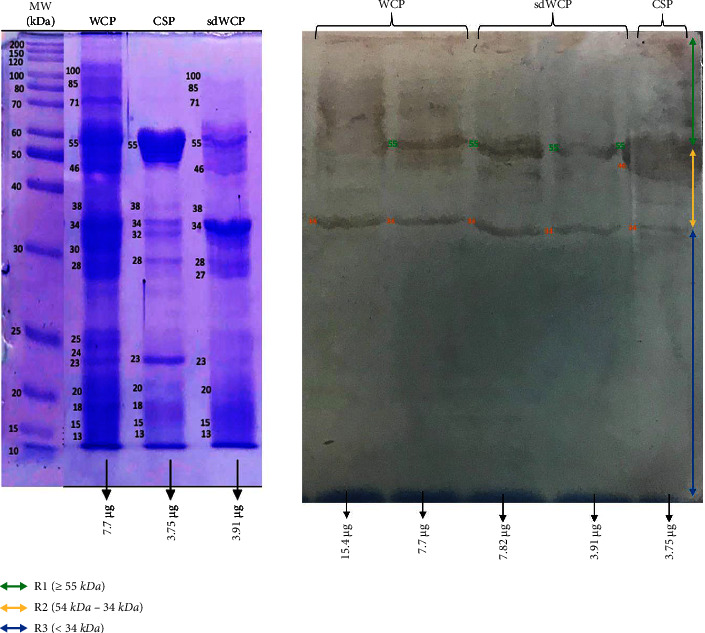
The WB analysis of IgA antibody isotype reactivity against SDS-PAGE-separated antigens from the WCP, CSP, and sdWCP of *S.* spp against PHPS. The immunoreactive bands identified in the WB reaction are classified according to the region (top right) and indicated by coloured arrows on the right. The reference lane molecular weight markers along with the protein profile stained with Coomassie are indicated to the left most.

WB analysis was also performed to analyze the reactivity of the panel of pooled sera from TYPHOIDYNE EIA test positive healthy subjects designated as PHPS against the differentially extracted antigens derived from *S. typhi* and *S.* spp. As shown in Figures 9–14, the WB profiles of reactive bands against IgM, IgG, and IgA antibody isotypes were largely confined in cluster 1 and cluster 2. The differentially extracted *S. typhi* and *S.* spp antigens in cluster 3 were not recognized by the PHPS serum pool against all three antibody isotypes.

On the other hand, significant difference was observed when comparing the WB profiles of PTS and PHPS. The WB profiles of the differentially extracted antigens derived from *S. typhi* and *S.* spp against IgM, IgG, and IgA antibody isotypes in the PTS sera showed stronger reactivity in terms of the degree of intensity and number of protein bands compared to PHPS sera. The summary of the overall WB reactivity profiles produced by the pooled sera from typhoid patients and possible carriers is summarized in Tables 2–4. Based on Tables 3 and 4, reactive low molecular weight bands were not detected against IgG and IgA antibody isotypes in the WB assay for both PTS and PHPS serum pool.

## 4. Discussion

The differentially extracted antigens derived from *S. typhi* and *S.* spp were also classified according to the somatic (O9) and flagellar (dH) antigens by WB assay. The pattern of recognition in WB profiles probed with O9 and dH serum was analyzed to further understand the significance of these antigens in the serodiagnosis of typhoid fever. Based on Figures 1 and 2, the somatic and flagellar antigens observed in the WB assay were heterogenous and generally recognized by differentially extracted proteins derived from *S. typhi* and *S.* spp in PTS- and PHPS-pooled serum against all three antibody isotypes. Based on this finding, it is interesting to note that the differentially extracted crude proteins provided a moderate sense of specificity in the WB assay, particularly when variants are present. Due to the complexity of differentially extracted crude protein, a better separation technique such as liquid phase preparative isoelectric focusing (IEF) can be used to further identify the location of somatic and flagellar antigens.

To further analyze the host humoral immune response in typhoid patients against the multiantigen consisting of WCP, CSP, and sdWCP derived from *S. typhi* and *S.* spp, WB analysis was performed with typhoid sera and possible carrier sera. The sera from typhoid patients which tested positive in IgM, IgG, and IgA antibody isotypes in TYPHOIDYNE EIA were selected and tested by the WB assay in pooled samples. Healthy sera that were tested positive and categorized as possible carrier in TYPHOIDYNE EIA from our previous report were selected and pooled for the WB assay evaluation [14]. Comparative analysis of the multiantigen profile recognized by IgM, IgG, and IgA antibody isotypes in the WB assay probed with PTS and PHPS was performed. The use of pool sera is to minimize the variation of the reactivity in IgM, IgG, and IgA antibody isotypes against the multiantigens derived from *S. typhi* and *S.* spp as antigen recognition by individual sera which are varied [16, 17]. These two pools of sera were selected for WB assay in order to discriminate and define the profiles of reactive antigenic bands between differentially extracted antigens produced by each antibody isotypes in typhoid patients compared to healthy subjects with a possible history of exposure to typhoid fever.

Based on the results, we identified up to 16 distinct antigenic bands with molecular weights ranging from 100 kDa to 20 kDa in the WB assay probed with PTS and PHPS against three antibody isotypes with various degree of intensity and frequency at a given concentration. These antigenic bands were categorized into three main clusters of antigens consisting of broadly diffused antigenic bands (RI), regularly spaced antigenic bands (R2) and low molecular weight antigenic bands (R3). Based on the WB assay results for PTS, the IgM, IgG and IgA antibody isotypes elicited strong reactivity to all three differentially extracted antigens derived from both *S. typhi* and *S.* spp majorly in cluster 1 and 2. These results indicated that the highly reactive antigens in cluster 1 and 2 were responsible in triggering strong immune response from all three antibody isotypes in typhoid infection. This finding also demonstrated that closely related *Salmonella* species share number of antigens with S. typhi, which may indicate the cross-protective roles of these antigens.

On the other hand, WB assay probed with PHPS against IgM, IgG, and IgA antibody isotypes expressed similar antigenic reactivity as PTS with the reactive bands from *S. typhi* and *S.* spp largely confined in cluster 1 and 2. As the protein in cluster 1 and 2 in both serovars were recognized by PHPS in this study, it is highly likely that these proteins are conserved protein among *Salmonella* and are able to stimulate the immune response among both typhoid patients and healthy subjects suggesting that they could be good vaccine candidates. However, the degree of antigenic intensity between PTS and PHPS can clearly be differentiated as PTS elicited a stronger degree of antigenic intensity compared to PHPS. Overall, the diverse degree of antigenic intensity of the bands between PTS and PHPS highlighted the heterogenicity in the humoral immune response in typhoid patients and healthy subjects with a possible history of exposure to typhoid fever.

In the subsequent review of the WB results, we compared the reactivity of multiantigens against IgM, IgG, and IgA antibody isotypes to obtain a distinct fingerprint pattern in the WB assay. We observed that PTS patients and PHPS subjects both produced markedly heterogenous reactivity against the IgM, IgG, and IgA antibody isotypes response with respect to the intensity, number, and location of the bands.

IgM, which is the initial antibody isotype produced during an immune response, serves as the body's main defense against infections [18]. In the previous study, the role of IgM as an indicator for acute infection of *M*. *p*n*eumonia* has been well documented [19]. These findings is in line with the results we have achieved in our study whereby the PTS serum pool produced profiles of diffuse bands with visible background reaction in clusters 1 and 2 based on the overall view of the humoral immune response in IgM antibody isotype of PTS and PHPS against the genus conserved and species-specific antigens derived from *S. typhi* and *S.* spp in Table 2. In addition, most of the low molecular weight antigenic bands derived from both *S. typhi* and *S.* spp were recognized by IgM antibody isotypes in PTS. Surprisingly, WB assay performed with the PHPS serum pool revealed no visible IgM antibody isotypes reactive bands in cluster 3. Previous data analysis report on the Typhidot diagnostic test has revealed the high avidity of IgM antibody in the detection of acute typhoid infection [20]. Several authors have suggested that IgM antibody isotypes are mostly generated during the early stages of infection [21–23]. IgM antibodies have been shown to identify a variety of microbial components, including viral antigens and bacterial toxins [24]. As mentioned in the previous study, IgM may remain for a very long time; hence, this antibody isotype can still be detected in carriers but at a lower degree of intensity.

There have been frequent reports on the persistence of IgG antibodies for more than two years following infection. In addition, IgG antibodies that were generated later than IgM antibodies frequently persist for a lifetime [25–27]. Our findings extent these previous evaluations significantly as we observed a relatively similar pattern of humoral immune response in IgG antibody isotype in both PTS and PHPS. Both categories of the subjects elicited well-separated antigenic bands at the location above 34 kDa. Although the location of the bands observed in the WB assay for IgG antibody isotypes was analogous, a clear difference in terms of the degree of antigenic intensity was demonstrated in the WB assay whereby PTS demonstrated a higher intensity of reaction compared to PHPS. In addition, a number of reports in the literature have delineated the presence and reactivity of *Salmonella* flagellin antigen with the expected molecular weight of 55 kDa [28–30]. In this study, the 55 kDa antigen was also detected in PTS- and PHPS-pooled serum against the IgG antibody isotype. The presence of reactive 55 kDa antigenic band in the WB profiles of both typhoid and possible carrier subjects suggests the role of this flagellin antigen as a marker for protective immunity.

Based on the WB assay result for the IgA antibody isotype, we remarked a certain degree of homogenous antigenic protein pattern with two major bands recognized in both PTS and PHPS at the location of 34 and 55 kDa. In the previous study, the antigenic protein with the molecular weight of 34 kDa has been identified, purified, and characterized as cytolysin, a cytotoxic protein from the outer membrane of *S*. *typhi*. The utility of 34 kDa cytotoxin protein against the IgA antibody has been well demonstrated as a diagnostic biomarker for typhoid fever [31, 32].

On the other hand, the WB profile of PHPS recognized the IgA antibody isotype antigenic band at the region of 32 kDa which was not seen in other antibody isotypes. In addition, no visible background reaction was detected in IgA antibody isotypes, which indicated a high specificity for IgA antibody isotype reaction against the multiantigens characterized by the WB assay. Emerging data also highlighted the role of the IgA antibody isotype as a good biomarker of acute stage typhoid [33, 34].

Based on the WB assay, additional reactive bands located at different location in IgM, IgG, and IgA antibody isotypes were spotted and are described in Tables 2–4. Generally, we observed a clear distinguishment of the reactive multiantigen band profiles for IgM, IgG, and IgA antibody isotypes probed with PTS and PHPS. High intensities of reaction were observed particularly in IgM followed by IgA and IgG antibody isotypes of PTS. On the other hand, the degree of intensity of the reactive bands produced by PHPS serum pool in WB assay were lower and can be distinguished from the reactive bands found in the PTS serum pool. Based on the results in our study, we found out that the pattern of the humoral immune response in all three antibody isotypes were diversified and served as a potential fingerprint profile that could be conclusive for the serodiagnosis of typhoid fever.

We also noted the discrepancies between the reactivity of antigens exhibited between closely related *Salmonella* species which include *S. typhi* and *S.* spp in the WB assay. PTS pool sera recognized a greater number of antigenic bands derived from *S. typhi* compared to *S.* spp. Subsequently, PHPS pool sera recognized several reactive multiantigens derived from *S. typhi* and *S.* spp that were not present in PTS pool sera.

The production of several antigens at various stages of infection in malaria is also linked to the diversity of immune responses by *Plasmodium* spp. [35]. On the other hand, *M. Tuberculosis* is associated with the expression of antibodies against a range of antigens in most active TB patients [36]. Our findings extent these reports whereby the WB assay displayed a dynamic pattern of recognition with clear discrepancies in the number of antigenic bands present between three differentially extracted arrays of antigens derived from *S. typhi* and its closely related species. Overall, the arrays of antigens derived from *S. typhi* and *S.* spp successfully differentiated typhoid patients from healthy subjects with possible prior exposure to typhoid fever.

## Figures and Tables

**Table 1:** Description of the pooled sera and the designated codes for characterization by WB assay.

Designated codes	Description of the pooled sera	TYPHOIDYNE EIA results	No. of sera pooled
PTS	Culture positive typhoid cases	Positive	4
PHPS	Healthy subjects	Positive as the carrier	4

**Table 2:** Summary of the overall view of the humoral immune response in the IgM antibody isotype of PTS and PHPS against the antigens in the WCP, CSP, and sdWCP of *S. typhi* and *S.* spp analyzed by WB assay.

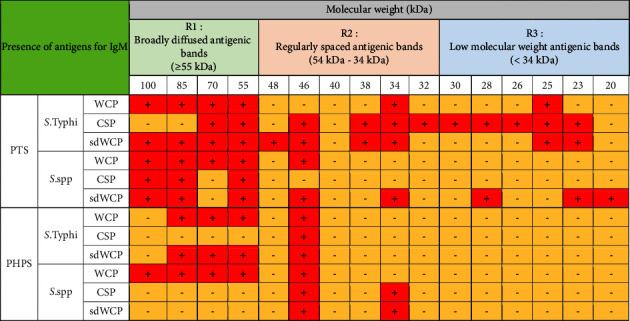

Note: The red-coloured box with the symbol “+” indicates the presence of immunoreactive bands, and the yellow-coloured box with the symbol “−” indicates the absence of an immunoreactive band.

**Table 3:** Summary of the overall view of the humoral immune response in IgG antibody isotype of PTS and PHPS against the antigens in the WCP, CSP, and sdWCP of *S. typhi* and *S.* spp analyzed by WB assay.

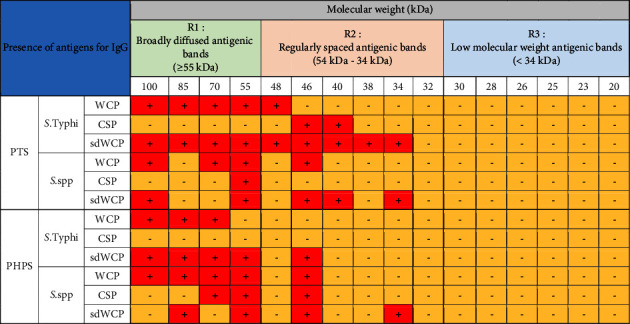

Note: The red-coloured box with the symbol “+” indicates the presence of immunoreactive bands, and the yellow-coloured box with the symbol “−” indicates the absence of an immunoreactive band.

**Table 4:** Summary of the overall view of the humoral immune response in IgA antibody isotype of PTS and PHPS against the antigens in the WCP, CSP, and sdWCP of *S. typhi* and *S.* spp analyzed by WB assay.

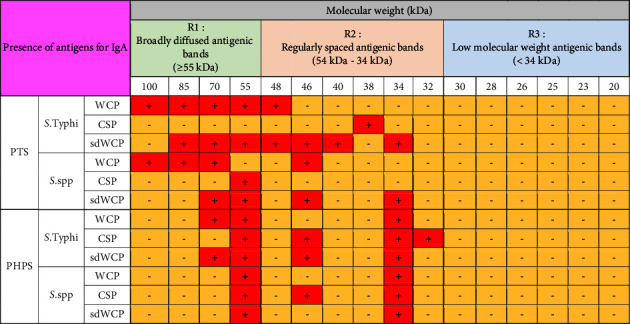

Note: The red-coloured box with the symbol “+” indicates the presence of immunoreactive bands, and the yellow-coloured box with the symbol “−” indicates the absence of an immunoreactive band.

## Data Availability

All data used to support the findings of this study are included within the article.
